# Incidence and predictors of common opportunistic infection among HIV -infected children attending antiretroviral treatment clinic at Northeast Ethiopia, public hospitals 2022: A multicenter retrospective follow-up study

**DOI:** 10.1016/j.amsu.2022.104910

**Published:** 2022-11-16

**Authors:** Endalk Birrie Wondifraw, Birhanu Desu Tefera, Mulusew Zeleke, Samuel Nebyu, Lehulu Tilahun, Mulugeta W/Selassie, Zenebe Tefera

**Affiliations:** a Department of Pediatric and Child Health Nursing, College of Medicine and Health Science, Wollo University, Dessie, Ethiopia; b Department of Emergency and Critical Care Nursing, College of Medicine and Health Science, Wollo University, Dessie, Ethiopia; c Department of Adult Nursing, College of Medicine and Health Science, Wollo University, Dessie, Ethiopia; d Department of Midwifery, College of Medicine and Health Science, Wollo University, Dessie, Ethiopia

**Keywords:** Incidence, Opportunistic infections, Children, Anti-retroviral therapy, Northeast, Ethiopia

## Abstract

**Background:**

Opportunistic infections (OIs) are illnesses that attack people with weakened immune systems, such as HIV patients, more frequently and severely. The majority of opportunistic infections (OIs) are the leading causes of morbidity and mortality in HIV/AIDS patients, emerging at the end of the illness. The objective of this study was to assess the incidence and risk factors of opportunistic infections (OIs) in HIV-infected children receiving antiretroviral therapy in public hospitals in Northeast Ethiopia.

**Methods:**

A multicenter retrospective follow-up study was undertaken at public hospitals in northeast Ethiopia from September 1, 2010, to January 30, 2022. A total of 341 HIV-infected children on antiretroviral therapy were included in the study. Data was entered using Epi-Data Manager version 4.6.1, and it was analyzed using STATA version 16.1. The opportunistic infection free-survival time was estimated using the Kaplan-Meier survival curve. Bivariable and multivariable Cox proportional hazard models were used to investigate the determinants of opportunistic infections.

**Results:**

The overall incidence rate of opportunistic infections (OIs) was 6.0 (95% CI: 5.0–7.1) per 100 child-years of observation. This study's participants were observed for a minimum of 9 months and a maximum of 122 months, for a total of 21,629 months, or 1802.4 years. Children with WHO clinical stages III and IV (AHR: 1.77; 95% CI: 1.13, 2.77), non-users of Cotrimoxazole Preventive Therapy (CPT) (AHR: 2.10; 95% CI: 1.40, 3.08), and low hemoglobin levels (10 mg/dl) (AHR: 1.88; 95% CI: 1.25, 2.82) were identified as significant predictors of opportunistic infection.

**Conclusion:**

In this study, the incidence rate of opportunistic infections among HIV-infected children was found to be high when compared to other studies. Low hemoglobin levels (10 mg/dl), low CD4 counts or percentages, clinical stages III and IV, and non-users of CPT were all associated with higher rates of opportunistic infection.

## Highlights

• Opportunistic infections (OIs) are infections that occur more frequently.
• Opportunistic infections (OIs) are more severe in people with weakened immune systems.
• Opportunistic infections (OIs) are main causes of morbidity and mortality in people with HIV.

## 1 Introduction

Opportunistic infections (OIs) are illnesses that attack people with weakened immune systems, such as HIV patients, more frequently and severely [1,2]. Clinical manifestations of HIV infection range from early infection and a long period of asymptomatic status to advanced disease [[1], [2], [3]]. Opportunistic infections account for the majority of morbidity and mortality in HIV/AIDS patients, emerging at the end of their illness [3].

It is one of the major causes of morbidity and mortality in people with HIV [4]. Children in particular bear a significant burden throughout their final stages [3]. Prior to the creation of highly effective combination antiretroviral treatment (HAART) regimens during the antiretroviral era, opportunistic infections (OIs) were the main causes of death in children with human immunodeficiency virus (HIV) infection [5]. Both in adults and children, the prevalence of AIDS-related OIs and mortality has considerably increased and dramatically decreased as a result of current HAART regimens, which also significantly and significantly provide immunological reconstitution. However, mortality remains high, particularly in developing countries [2,6,7].

In 2019, 1.63 million children in middle- and lower-income countries are expected to have been tested, up from 1.59 million in 2018. According to some estimates, this amounts to approximately 60% coverage, though it does not provide a complete picture. Over 160,000 infants have been diagnosed with HIV, and over 100,000 deaths have been attributed to AIDS, indicating that morbidity and mortality remain unacceptably high [3].

In urban Ethiopia, approximately 19,000 children tested positive for HIV, representing 0.3% prevalence [2]. Opportunistic infections have a significant impact on the mortality of HIV/AIDS-affected children. Despite the fact that opportunistic infections are a significant cause of morbidity and mortality in HIV-infected children, research on these infections is scarce, particularly in the study area.

Children with HIV who have opportunistic infections are at an advanced stage of the disease, and the majority of these infections frequently result in the death of infection victims [1,8]. Almost 90% of HIV/AIDS deaths are caused by opportunistic infections and cancer. According to studies, people living with HIV/AIDS are susceptible to a variety of opportunistic infections [6].

In Ethiopia, OI diagnosis and treatment have been linked with HIV care in order to prevent, identify, and treat OI in HIV-positive children. However, in order to do so, a thorough understanding of the environment is required, particularly in the research location where the incidence rate of OIs and its associated determinants in young people living with HIV has not been thoroughly investigated.

The objective of this study was to assess the incidence and risk factors of opportunistic infections (OIs) in HIV-infected children receiving antiretroviral therapy in public hospitals in Northeast Ethiopia.

## 2 Methods and materials

### 2.1 Study design, setting, and period

A multicenter retrospective follow-up study was conducted at public hospitals in northeast Ethiopia from September 1, 2010 to January 30, 2022. Dessie and Woldia Comprehensive Specialized Hospitals are involved. The Dessie Comprehensive Specialized Hospital is located in Dessie Town. This is the South Wollo Zone's capital. It is approximately 400 km from Addis Abeba, Ethiopia's capital city. The hospital serves approximately 5.5 million people. The ART service was launched in 2005. The Woldia Comprehensive Specialized Hospital is located in Woldia Town, the largest city in the North Wollo Zone. It is 520 km from Addis Abeba, the capital. As a referral hospital, this hospital serves nearly 4 million people.

### 2.2 Source population

All HIV infected children at northeast Ethiopia's public hospitals, 2022.

### 2.3 Study population

From September 1, 2010, to January 30, 2022, all HIV-infected children under the age of 15 in northeast Ethiopia received ART in public hospitals.

### 2.4 Inclusion criteria

From September 1, 2010, to January 30, 2022, all HIV-infected children under the age of 15 who received ART for at least one month are eligible.

### 2.5 Exclusion criteria

Children with common opportunistic infections at baseline, children with incomplete chart recording at baseline and during the follow-up period, particularly critical information such as ART regimen, date of ART initiation, and date of incident, or children who reported incidents that were censored, were also excluded from the study.

### 2.6 Sample size determination and sampling procedure

The sample size was determined using a log-rank survival data analysis of the two-population proportion calculation. In addition, the study's “Fair” and “poor” adherence levels were used as the exposed group, denoted by q1 (0.59), and “Good” adherence levels were used as the non-exposed group, denoted by q0 (0.48) from a study that was conducted at Debre Tabor referral Hospital and University of Gondar Compressive specialized hospitals [8], and the total final sample size, after adding 10 incomplete data was 354. 354 samples were selected randomly by lottery method among 2036 ART users who started ART between September 1, 2010, to January 30, 2022.

### 2.7 Data collection tools and procedures

The Federal Ministry of Health's HIV-care/ART follow-up and intake records were used to adapt the data collection checklist. The data extraction form included socio-demographic information, ART and other drugs, clinical and laboratory data, and more. Four BSc nurses with ART service experience and training collected data over the course of four weeks in February 2022, under the supervision of two MSc nursing practitioners.

### 2.8 Data quality assurance

A week before beginning data collection, the principal researcher completed a pretest on 18 (5%) randomly selected charts at Dessie Comprehensive Specialized Hospital to ensure the consistency and clarity of the instrument. Supervisors and data collectors were trained for two days on the types of data that should be collected and how to obtain relevant data. Every day, the main investigator and supervisor double-checked the collected data to ensure its accuracy. Charts with missing data from the data collection process were not included.

## 3 Variables

**Dependent variable:** common opportunistic infections.

### 3.1 Independent variables

#### 3.1.1 **Socio-demographic characteristics**: age, sex

**Clinical, laboratory, and medication-related characteristics:** WHO clinical staging, HIV disclosure status, Weight for age, height for age, hemoglobin level, CD4 (Cluster of Differentiation 4) counts or %, regimen at baseline, Cotrimoxazole preventive therapy (CPT), Isoniazid preventive therapy (IPT), level of adherence to ART, and duration on ART.

### 3.2 Operational definition

**Event**: common opportunistic infections occurring during the observation period.

**Time to develop common opportunistic infection**: The time from children's ART initiation to the occurrence of common opportunistic infection during the observation period.

**Common opportunistic infections**: They are a serious form of opportunistic infection that affects people with weaker immune systems, such as those who have the human immunodeficiency virus, more frequently and severely (HIV). Some of the most common OIs include bacterial pneumonia, pulmonary tuberculosis, extra pulmonary tuberculosis, oral and esophageal candidiasis, chronic diarrhea that lasts longer than a month, pneumocystis pneumonia, toxoplasmosis, Cryptococci meningitis, non-lymphoma, Hodgkin's Kaposi's sarcoma, wasting syndrome, and other OI [9].

**Censored**: Lost, drop out, transfer out, died of other causes or completed study period before developing opportunistic infection.

**Adherence**: Based on the percentage of drug dosage determined from the total monthly dosages of ART medications taken, we categorized adherence to ART into good, fair, and poor (Good >95%, fair 85–94%, poor >85%) [10].

**Underweight or stunting**: according to WHO growth curve weight/age < −3 z score and height/age < −3 z [11].

**CD4 cell count**: The CD4 cell count below the threshold level was divided into four categories based on the child's age: CD4 cell count 1500/mm3 (25%) for 12 months, CD4 cell count 750/mm3 (20%) for 12–35 months, CD4 cell count 350/mm3 (15%) for 36–59 months, and CD4 cell count 200/mm3 (15%) for 60 months [12].

### 3.3 Data processing and analysis

Data were reviewed for consistency, coding errors, completeness, accuracy, clarity, and missing values before being entered into Epi-Data Manager version 4.6.1 and analyzed by STATA version 16.0 Software. With the use of the median, mean, proportion, frequency, and interquartile range, descriptive and summary statistics were computed. Tables and graphics were used to present the data. The median time to frequent opportunistic infections over the follow-up period was estimated using the Kaplan-Meier curve, and log-rank tests were used to evaluate survival curves between various categories of predictive variables. The number of children who acquired common OIs throughout the follow-up period was divided by the number of person-years the children were under observation to determine the incidence of common opportunistic illnesses. The Schoenfeld residuals test (global test = 0.85200) was used to verify the basic premises of the Cox proportional hazard regression model, and the Cox-Snell residual was compared to the cumulative hazard function to determine the model's fit. To find predictors of typical opportunistic infections, the Cox proportional hazard model was fitted to both bivariable and multivariable data. To identify a significant variable, the bivariable analysis variables with a p-value of up to 0.25 were added to the multivariable model. Variables with p-values < 0.05 were regarded as statistically significant predictors of typical OIs in the final model. The presence and degree of correlations were summarized using an adjusted HR (AHR) with 95% confidence intervals.

## 4 Result

### 4.1 Socio-demographic characteristics of the children

All 354 HIV-infected children's medical records were obtained. The study of 341 medical records of HIV-infected children receiving ART yielded a completeness rate of 96.3%. The median age of the study participants was 8 years (IQR = 4.5, 10). Two-thirds of 237 (69.5%) children were over 10 years old. More than half of the 180 (52.8%) children were males. The vast majority of children (83.6%) resided in cities, and 88.3% of them lived with their parents. Twenty-nine (8.5%) were government employees by occupation (Table 1).

**Table 1:** Socio-demographic characteristics of HIV-infected children on antiretroviral therapy at northeast Ethiopia public Hospitals, 2022.

Characteristics		Frequency (n = 341)	Percentage
Age of the chid (years)	<5 years	63	18.5
	5–9 years	41	12.0
	≥10 years	237	69.5
Sex	Male	180	52.8
	Female	161	47.2
Residence	Urban	285	83.6
	Rural	56	16.4
Relation of the caregiver to the child	Parent	301	88.3
	Sister/brother	22	6.5
	Uncle/aunt	8	2.3
	Grandparent	10	2.9
Marital status of caregiver	Single	12	3.5
	Married	224	65.7
	Divorced	13	3.8
	Widowed	92	27.0
Caregiver's occupation status	House wife	77	22.6
	Governmental employee	29	8.5
	Non-governmental employee	195	57.2
	Merchant	21	6.2
	Farmer	19	6.6

### 4.2 Clinical, laboratory, and medication-related characteristics

CD4 counts, or percentages above the cutoff, were present in slightly more than half (53.1%) of the children. Two-thirds (71.3%) of children had a good degree of adherence to ART during the follow-up period, and around half (54.3%) of children were categorized as WHO clinical stages I and II. Seventy-two (21.1%) of the children experienced drug-related side effects, and 61.6% of the children had hemoglobin levels below 10 mg/dl. Furthermore, 72.7% and 45.7% of children used IPT and CPT, respectively. Approximately 89.4% of children took ART for more than 34 months, whereas 16.1% had unsuccessful medical therapy (Table 2).

**Table 2:** Clinical, laboratory and treatment-related characteristics of HIV-infected children on antiretroviral therapy at northeast Ethiopia public Hospitals, 2022.

Characteristics		Frequency (n = 341)	Percentage
Treatment failure	Yes	55	16.1
	No	286	83.9
Drug side effect	Yes	72	21.1
	No	269	78.9
CD4 counts or % level	Below threshold	160	46.9
	Above threshold	181	53.1
WHO clinical staging	I/II	185	54.3
	III/IV	156	45.7
IP	Given	248	72.7
	Not given	93	27.3
CPT	Given	156	45.7
	Not given	185	54.3
Weight for age	Normal	292	85.6
	Underweight	49	14.4
Height for age	Normal	300	88.0
	Stunting	41	12.0
Adherence	Good	243	71.3
	Fair/Poor	98	28.7
Duration of follow-up in months	<34 months	36	10.6
	>34 months	305	89.4
Opportunistic infections	Yes	130	38.1
	NO	211	61.9
Hemoglobin level	<10 mg/dl	131	38.4
	≥10 mg/dl	210	61.6
Initiation regimen	EFV based	236	69.2
	NVP,PI and other based	105	30.8

### 4.3 Incidence of common opportunistic infections during follow-up

**Fig. 1:**
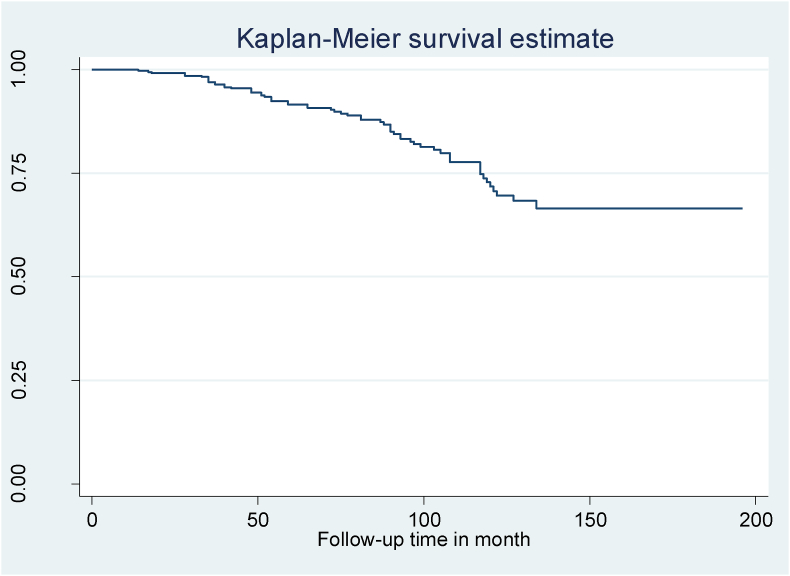
Kaplan-Meier of common Opportunistic infection-free survival time among HIV infected children on ART at northeast Ethiopia's public hospitals, from September 1, 2010, to January 30, 2022.

In this study 38.1% of children develop common OIs. The total OIs incidence rate was 6.0 (95% CI: 5.0–7.1) per 100 child-years of observation. Participants in this study were observed for a minimum of 9 months and a maximum of 122 months, giving a total of 21,629 months or 1802.4 years of observation (Fig. 1). The median OIs-free survival duration was 97 months (IQR = 66, 117) in this study. The most frequent condition seen in 32.3% of the children was tuberculosis, which was followed by bacterial pneumonia (16.1%) and herpes zoster (12.3%) (Table 3).

**Table 3:** Common types of OIs during follow-up time among HIV-infected children on antiretroviral therapy at northeast Ethiopia public Hospitals, 2022.

Common types of OIs	Frequency	Percentage
Herpes zoster	16	12.3
PGL	7	5.4
Candidiasis	17	13
Diarrhea	8	6.1
Pneumonia	21	16.1
purpratic reaption	7	5.4
TB	42	32.3
Herpes simplex	9	6.9
Others	3	2.3

Note - PGL: Persistence generalized lymphadenopathy.

### 4.4 Common opportunistic infections free survival time of predictor variable

**Fig. 2:**
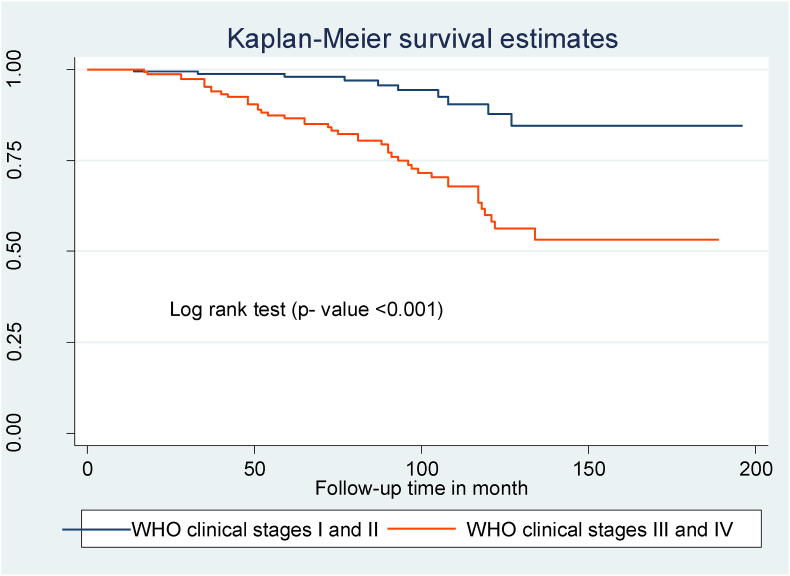
Kaplan Meier survival curve of WHO clinical stages among HIV- infected children on ART at northeast Ethiopia's public hospitals 2022.

**Fig. 3:**
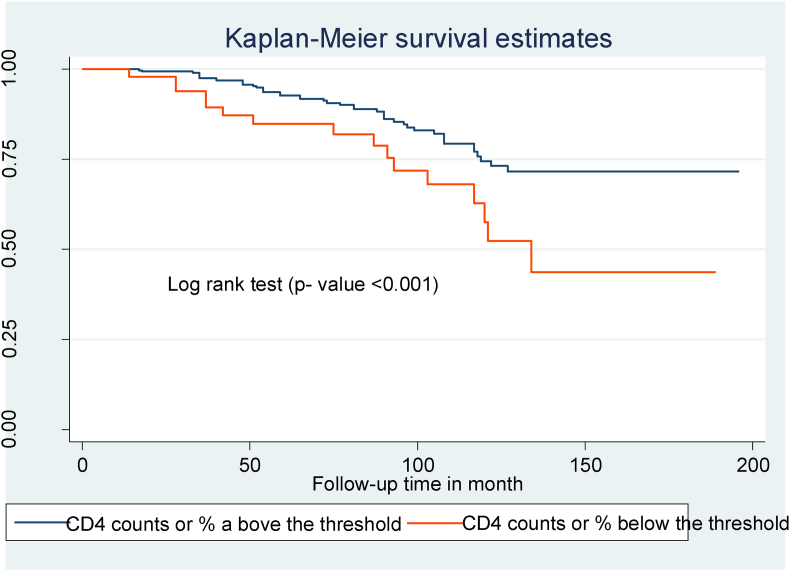
Kaplan Meier survival curve of CD4 count or percent among HIV- infected children on ART at northeast Ethiopia's public hospitals 2022.

**Fig. 4:**
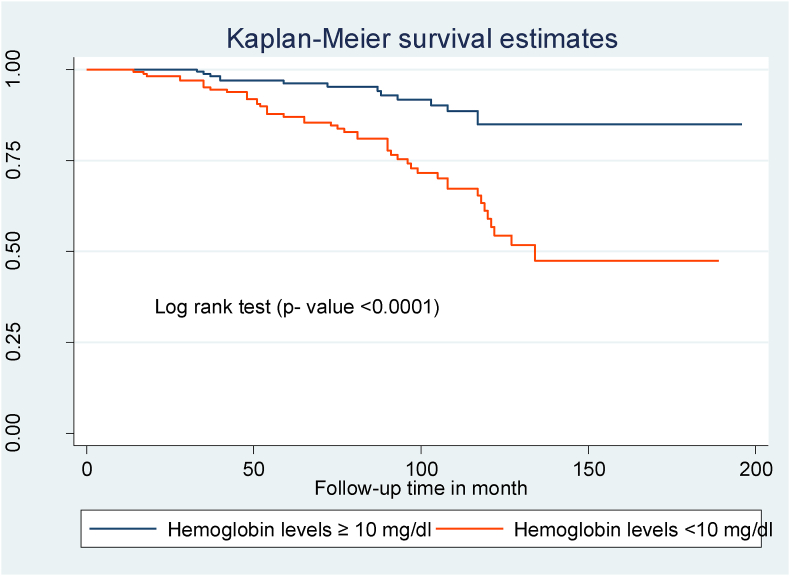
Kaplan Meier survival curve of hemoglobin levels among HIV- infected children on ART at northeast Ethiopia's public hospitals 2022.

**Fig. 5:**
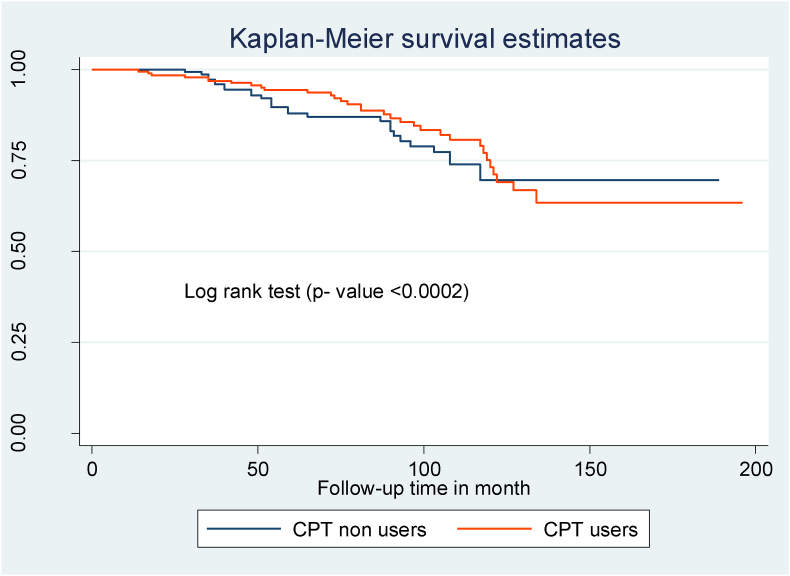
Kaplan Meier survival curve of Cotrimoxazole preventive therapy among HIV- infected children on ART at northeast Ethiopia's public hospitals 2022.

Compared to children with WHO clinical stages I and II, those with WHO clinical stages III and IV at the start of ART had a shorter OIs-free survival time (Fig. 2). Children with mild immunodeficiency (CD4 count or percent above the threshold) had a longer OIs-free survival time than children who presented with severe immunodeficiency (CD4 count or percent below the threshold) (Fig. 3). Children with low hemoglobin levels (<10 mg/dl) and those who did not take CPT also had shorter free OIs-free survival times than their counterparts (Fig. 4) and (Fig. 5).

### 4.5 Predictors of common opportunistic infections

CD4 count or %, WHO clinical staging, Duration of follow-up in months, weight for age, taking IP prophylaxis, disclosure status, level of hemoglobin, level of adherence, taking CPT prophylaxis, and initiation regimen were variables included in the multivariable analysis. WHO clinical stages III and IV, CD4 levels or percentages below the threshold, CPT non-users, and low hemoglobin levels (<10 mg/dl) were discovered to be important predictors of OIs. The risk of having OIs was 1.77 times higher in children with WHO clinical stages III and IV (AHR: 1.77; 95% CI: 1.13, 2.77) than in children with WHO clinical stages I and II. Children with CD4 counts or percentages below the criteria were 2.53 times (AHR: 2.53; 95% CI: 1.65, 3.87) more likely to have OIs than children with CD4 counts or percentages above the threshold. Children who did not utilize CPT were 2.1 times (AHR: 2.10; 95% CI: 1.40, 3.08) more likely to acquire OIs than those who did use CPT. Finally, children with low hemoglobin levels (less than 10 mg/dl) were roughly 1.88 times (AHR: 1.88, 95% CI: 1.25, 2.82) more likely to develop OIs than children with normal hemoglobin levels (greater than 10 mg/dl) (Table 4).

**Table 4:** Cox-proportional hazard analysis of predictors of Common OIs among HIV-infected children on antiretroviral therapy at northeast Ethiopia public Hospitals, 2022.

Variables		Survival status		CHR (95% CI)	AHR (95% CI)
		Event	Censored		
Age of the child (years)	<5 years	38	25	1	–
	5–9 years	29	12	1.02(0.51–1.57)	–
	≥10 years	144	93	1.18(0.75–1.84)	–
Sex	Male	110	70	1.11(0.78–1.57)	–
	Female	101	60	1	–
CD4 count or %	Below threshold	64	96	3.36(2.27–4.97)	2.53(1.65–3.87)**
	Above threshold	147	34	1	1
WHO clinical staging	I/II	155	30	1	1
	III/IV	56	100	3.47(2.30–5.23)	1.77(1.13–2.77)*
IP	Given	153	95	1	1
	Not given	58	35	1.37(0.92–2.04)	0.95(0.62–1.45)
CPT	Given	122	34	1	1
	Not given	89	96	2.53(1.71–3.74)	2.10(1.40–3.08)*
Hemoglobin level	<10 mg/dl	41	90	3.14(2.16–4.57)	1.88(1.25–2.82)**
	≥10 mg/dl	170	40	1	1
Weight for age	Normal	187	105	1	1
	Underweight	24	25	1.37(0.88–2.13)	1.17(0.72–1.90)
Height for age	Normal	189	111	1	–
	Stunting	22	19	1.08(0.66–1.76)	–
Disclosure status	Disclosed	102	72	1	1
	Non disclosed	109	58	1.22(0.86–1.72)	0.78(0.54–1.13)
Adherence	Good	162	81	1	1
	Fair/Poor	49	49	1.84(1.29–2.63)	1.45(0.97–2.18)
duration on ART	<34 months	27	9	1	1
	>34 months	184	121	3.57(1.77–7.20)	1.20(0.93–3.44)
Initiation regimen	EFV based	65	40	1.38(0.94–2.00)	1.31(0.89–1.94)
	NVP,PI and other based	146	90	1	1

**Notice** - *Significant at <0.05 ** Significant at <0.01; CHR: Crude hazard ratio; AHR: adjusted hazard ratio; 1: reference category; CI: confidence interval CPT: cotrimoxazole prophylactic therapy; IPT: isoniazid prophylactic therapy; HGB.

## 5 Discussion

A multicenter retrospective follow-up study was conducted in northeast Ethiopian public hospitals to investigate the incidence and predictors of common opportunistic illnesses among HIV-infected children on ART.

In this study, the median common OIs-free survival time was 97 months, and the overall incidence rate was 6.0 (95% CI: 5.0–7.1) per 100 child-years of observation among HIV-infected children in public hospitals in northeast Ethiopia. This finding is consistent with research was undertaken in Northwest Ethiopia (5.3 per 100 person-years) [8], Italy (6.76 per 100 person-years) [13], and the United States(4.99 per 100 person-years) [14]. However, the incidence of common OIs discovered in this study is higher than that seen in Latin America (1.1 per 100 person-years) [15] and Brazil (2.63 per 100 person-years) [16]. Furthermore, this disparity may be explained by the fact that affluent countries have more sophisticated procedures for the early diagnosis, treatment, and management of OIs than resource-constrained settings such as Ethiopia, and the larger challenges of poverty, overcrowding, and malnutrition in developing countries may be a factor in the higher frequency of OIs among HIV-infected children.

Tuberculosis was the most common opportunistic illness (32.3%) during the follow-up period. This finding is consistent with studies conducted in Ethiopia (29.8%) and India (34.6%) [17,18]. In contrast, a study conducted in Ethiopia, North America, Latin America, and China discovered that pneumonia is a prevalent opportunistic infection [8,14,[19], [20], [21]]. Furthermore, in this study, chronic diarrhea accounted for 16.1% of all common OIs.

In this study, the probability of acquiring OIs was 1.77 times higher in children with WHO clinical stages III and IV than in children with clinical stages I and II. This outcome is consistent with research conducted in Ethiopia [17], India [22,23], and Asia [24]. It is explained by the fact that advanced disease weakens immunity, resulting in increased viral multiplication and higher loads of opportunistic infections.

Children with CD4 counts or percentages below the criteria had a 2.53 times (AHR: 2.53; 95% CI: 1.65, 3.87) higher risk of developing OIs than children with levels above the threshold. This discovery is consistent with research conducted in Ethiopia [17], Uganda [25], India [18], and Asia [24]. CD4 cells are crucial components of the immune system because they help the body fight infections. Therefore, any conditions that lower CD4 cell counts will weaken the immune systems of children with HIV who are vulnerable to the development of opportunistic infections.

The risk of OIs was 1.88 times higher in children with low hemoglobin levels than in children with normal hemoglobin levels. This research is backed up by studies undertaken in Ethiopia [8], Nigeria [26], and Uganda [25]. This is because low hemoglobin levels can have serious effects on those living with HIV, ranging from impaired productivity and performance to a relationship to the disease process and increased death [27].

Children who did not utilize CPT were 2.1 times more likely to develop OIs than those who did use CPT. This conclusion is consistent with research conducted in Ethiopia [8,17], Zambia [28], and Latin America [15]. Cotrimoxazole preventive therapy (CPT) is a realistic, inexpensive, and well-tolerated approach to employing cotrimoxazole interventions for HIV/AIDS patients to reduce HIV/AIDS-related comorbidities and deaths caused by various bacteria, fungi, and protozoa. Furthermore, the Ethiopian ART guideline recommends starting CPT early in HIV-positive children who will benefit from it in order to prevent OIs [9,29].

### 5.1 Limitation of the study

One of this study, limitations is its retrospective nature. As a result, clinically relevant predictor variables such as children's educational status and family economic status, as well as community hygiene practices and patients' and caregivers' awareness levels were omitted from this study.

## 6 Conclusion and recommendation

In this study, the incidence rate of opportunistic infections among HIV-infected children was found to be high when compared to other studies. Opportunistic infection rates were linked to low hemoglobin levels (<10 mg/dl), low CD4 counts or percentages, WHO clinical stages III and IV, and non-users of CPT. As a result, several tactics, approaches, and programs should be considered in order to lower the occurrence of opportunistic infections among HIV-positive children. It is particularly critical to stress the predictors of opportunistic infections among children revealed in this study. More research is needed to find additional factors impacting the high occurrence of opportunistic infections.

## Ethical approval

Wollo University's College of Medicine and Health Science, department of pediatrics, and child health nursing ethical review committee provided approval. The reference number for this letter was (PCHN-251/2022).

## Sources of funding

This study did not receive any specific grant from funding agencies in the public, commercial, or not-for-profit sectors.

## Author contributions

EBW, BDT, MZ, SN; participate in writing proposal, analyzed the data, wrote the result and discussion. LT, MW, ZT; participate in analyzing the data, writing result and prepared manuscript.

## Registration of research studies

1. Name of the registry: Research Registry.
2. Unique Identifying number or registration ID: Research Registry 8243.
3. Hyperlink to your specific registration (must be publicly accessible and will be checked): https://www.researchregistry.com/browse-the-registry#home/

## Guarantor

Corresponding author - Endalk Birrie.

You can contact the above guarantor to access the data.

## Consent

Each hospital administration and ART clinic focal person provided a permission letter. Since we were going to do chart reviews of secondary data, there was no need for informed consent. The consent was formally waived by the Ethics Committee.

## Provenance and peer review

Not commissioned, externally peer reviewed

## Declaration of competing interest

The authors declare no conflict of interest.

## Abbreviation

AHR: Adjusted hazard ratio
AIDS: Acquired immunodeficiency disease
ART: Antiretroviral therapy
CD4: Cluster of differentiation 4
CI: Confidence interval
CPT: Cotrimoxazole prophylactic therapy
HGB: Hemoglobin
HIV: Human immunodeficiency virus
IPT: Isoniazid prophylactic therapy
TB: Tuberculosis
OI: Opportunistic infection
PI: protease inhibitor
PYO: Person per year observation
WHO: World health organization
