# Supplementary material for: Incidence and predictors of common opportunistic infection among HIV -infected children attending antiretroviral treatment clinic at Northeast Ethiopia, public hospitals 2022: A multicenter retrospective follow-up study
Source: Ann Med Surg (Lond). 2022 Nov 16;84:104910. doi: 10.1016/j.amsu.2022.104910 (PMC9758350; doi:10.1016/j.amsu.2022.104910)
Supplement: Multimedia component 1 [file mmc1.docx]

| The STROCSS 2021 Guideline | | |
| --- | --- | --- |
| Item no. | **Item description** | **Page** |
| TITLE | | |
| 1 | **Title**  Incidence and predictors of common opportunistic infection among HIV -infected children attending ART clinic at Northeast Ethiopia, public hospitals 2022: A multicentre retrospective follow-up study  *STROCSS 2021 guidelines apply to cohort studies as well as other observational studies (e.g. cross-sectional, case-control etc.) | 1 |
| ABSTRACT | | |
| 2a | **Introduction** –  **Background**- Opportunistic infections (OIs) are illnesses that attack people with weakened immune systems, such as HIV patients, more frequently and severely. The majority of opportunistic infections (OIs) are the leading causes of morbidity and mortality in HIV/AIDS patients, emerging at the end of the illness.  **Scientific rationale for this study**- Even though OIs are a significant source of morbidity and mortality in children with HIV, studies on these infections are rare, especially in the study area.  **Aims and objectives**- This study's objective was to assess the prevalence and risk factors for OIs in HIV-infected children receiving ART in public hospitals in Northeast Ethiopia | 2 |
| 2b | **Methods** - From September 1, 2010, to January 30, 2022, a multicenter retrospective follow-up study was undertaken at public hospitals in northeast Ethiopia. A total of 341 HIV-infected children on ART were included in the study. Data was entered using Epi-Data Manager version 4.6.1, and it was analyzed using STATA version 16.1. The opportunistic infection free-survival time was estimated using the Kaplan-Meier survival curve. To investigate determinants of opportunistic infections, bivariable and multivariable Cox proportional hazard models were used. | 2 |
| 2c | **Results** - The total OIs incidence rate was 6.0 (95 % CI: 5.0-7.1) per 100 child-years of observation. Participants in this study were observed for a minimum of 9 months and a maximum of 122 months, giving a total of 21,629 months or 1802.4 years of observation.CD4 counts or % below the threshold (AHR: 2.53; 95% CI: 1.65, 3.87), Children presenting with WHO clinical stages III and IV (AHR: 1.77; 95% CI: 1.13, 2.77), Cotrimoxazole Preventive Therapy (CPT) non-user (AHR: 2.10; 95% CI: 1.40, 3.08), low and hemoglobin levels (<10 mg/dl) (AHR: 1.88, 95% CI: 1.25, 2.82) were discovered to be important predictors of Opportunistic infection. | 2 |
| 2d | **Conclusion** -In comparison to other research, the incidence rate of opportunistic infections among HIV-infected children was shown to be high in this study. Opportunistic infection rates were linked to low hemoglobin levels (<10 mg/dl), low CD4 counts or percentages, WHO clinical stages III and IV, and non-users of CPT. | 2 |
| INTRODUCTION | | |
| 3 | **Introduction**  **Relevant background and scientific rationale for study with reference to key literature:-** Opportunistic infections (OIs) are illnesses that attack people with weakened immune systems, such as HIV patients, more frequently and severely (1, 2). Clinical manifestations of HIV infection range from early infection and a long period of asymptomatic status to advanced disease (1-3). The majority of opportunistic infections (OIs) are the leading causes of morbidity and mortality in HIV/AIDS patients, emerging at the end of the illness (3).  It is one of the major causes of morbidity and mortality in people with HIV (4). Children in particular bear a significant burden throughout its final stages (3). Prior to the creation of highly effective combination antiretroviral treatment (HAART) regimens during the antiretroviral era, opportunistic infections (OIs) were the main causes of death in children with human immunodeficiency virus (HIV) infection (5). Both in adults and children, the prevalence of AIDS-related OIs and mortality has considerably increased and dramatically decreased as a result of current HAART regimens, which also significantly and significantly provide immunological reconstitution. However, mortality remains high, particularly in developing countries (2, 6, 7).  Worldwide, 1.63 million children in middle-class and lower-class nations are anticipated to have undergone testing in 2019, up from 1.59 million tests in 2018. This amounts to an estimated coverage of about 60%, according to some estimates, although it does not provide a complete picture. Over 160 000 infants have been reported to have HIV infection and over 100 000 deaths have been attributed to AIDS, indicating that morbidity and mortality remain unacceptably high (3).  In urban Ethiopia, there were 19,000 or so children who tested positive for HIV, which represents a prevalence of 0.3 percent (2). OIs significantly affect the mortality of HIV/AIDS-affected children. Even though OIs are a significant source of morbidity and mortality in children with HIV, studies on these infections are rare, especially in the study area.  Children with HIV who have OIs are at an advanced stage of the illness, and the majority of these infections frequently cause infection victims to pass away (1, 8). Opportunistic infections and cancers account for almost 90% of deaths from HIV/AIDS. Studies show that there are various opportunistic infections that affect persons with HIV/AIDS (6).  **Research question and hypotheses, where appropriate-** OIs diagnosis and treatment have been linked with HIV care in Ethiopia in order to prevent, identify, and treat OI in children living with HIV. But in order to do this, it's also crucial to have a complete understanding of the environment, particularly in the research location where the incidence rate of OI and its associated determinants in young people living with HIV has not been thoroughly examined.  **Aims and objectives-** This study's objective was to assess the prevalence and risk factors for OIs in HIV-infected children receiving ART in public hospitals in Northeast Ethiopia. | 3 & 4 |
| METHODS | | |
| 4a | **Registration**  This manuscript is registered in www.researchregistry.com with the unique identification number (UIN) of research registry 8243 (9).  ** “Every research study involving human subjects must be registered in a publicly accessible database before recruitment of the first subject”* | 4 |
| 4b | **Ethical approval**  Wollo University's College of Medicine and Health Science, department of pediatrics, and child health nursing ethical review committee provided approval. The reference number for this letter was (PCHN-251/2022). | 11 |
| 4c | **Protocol**   - Give details of protocol (*a priori* or otherwise) including how to access it (e.g. web address, protocol registration number etc.) - If published in a journal, cite and provide full reference | Not applicable |
| 4d | **Patient and public involvement in research**  The HIV-care/ART follow-up and intake records from the Federal Ministry of Health were used to adapt the data gathering checklist. The data extraction form contained information on socio-demographic factors, ART and other drugs, clinical and laboratory-related data, and more. Under the direction of two MSc nursing practitioners, four BSc nurses with ART service experience and training collected data.. | 5 |
| 5a | **Study design**  Multicentre retrospective follow-up study was undertaken at public hospitals in northeast Ethiopia. |  |
| 5b | **Setting and timeframe of research-** From September 1, 2010, to January 30, 2022, a multicenter retrospective follow-up study was undertaken at public hospitals in northeast Ethiopia. The hospitals involved are Dessie and Woldia Comprehensive Specialized Hospitals. Dessie Town is home to the Dessie Comprehensive Specialized Hospital. This is the capital of the South Wollo Zone. It is around 400 kilometers from Ethiopia's capital city, Addis Abeba. Around 5.5 million individuals are served by the hospital. The ART service began in 2005. The Woldia Comprehensive Specialized Hospital is located in Woldia Town, the North Wollo Zone's major city. It is 520 kilometers from the capital, Addis Abeba. This hospital serves almost 4 million people as a referral hospital. The data collected over the course of four weeks in February 2022. | 4 |
| 5c | **Study groups**   - Total number of participants - Number of groups - Detail exposure/intervention allocated to each group - Number of participants in each group | Not applicable |
| 5d | **Subgroup analysis** – comprehensively describe:   - Planned subgroup analyses - Methods used to examine subgroups and their interactions | Not applicable |
| 6a | **Participants** –  **Inclusion criteria**  From September 1, 2010, until January 30, 2022, all HIV-infected children under 15 years of age who took ART for at least one month qualify.  **Exclusion criteria**  Children with common opportunistic infections at baseline, children with incomplete chart recording at baseline and during the follow-up period, particularly critical information like ART regimen, date of ART commencement, and date of the incident were also excluded from the study . | 5 |
| 6b | **Recruitment** – 354 samples were selected randomly by lottery method among 2036 ART users who started ART between September 1, 2010, to January 30, 2022. | 5 |
| 6c | **Sample size** –The sample size was determined using a log-rank survival data analysis of the two-population proportion calculation. In addition, the study's "Fair" and "poor" adherence levels were used as the exposed group, denoted by q1 (0.59), and "Good" adherence levels were used as the non-exposed group, denoted by q0 (0.48) from a study that was conducted at Debre Tabor referral Hospital and University of Gondar Compressive specialized hospitals (8), and the total final sample size, after adding 10 incomplete data was 354. | 5 |
| METHODS - INTERVENTION AND CONSIDERATIONS | | |
| 7a | **Pre-intervention considerations** – comprehensively describe:   - Preoperative patient optimisation (e.g. weight loss, smoking cessation, glycaemic control etc.) - Pre-intervention treatment (e.g. medication review, bowel preparation, correcting hypothermia/-volemia/-tension, mitigating bleeding risk, ICU care etc.) | Not applicable |
| 7b | **Intervention** – comprehensively describe:   - Type of intervention and reasoning (e.g. pharmacological, surgical, physiotherapy, psychological etc.) - Aim of intervention (preventative/therapeutic) - Concurrent treatments (e.g. antibiotics, analgesia, anti-emetics, VTE prophylaxis etc.) - Manufacturer and model details, where applicable | Not applicable |
| 7c | **Intra-intervention considerations** – comprehensively describe:   - Details pertaining to administration of intervention (e.g. anaesthetic, positioning, location, preparation, equipment needed, devices, sutures, operative techniques, operative time etc.) - Details of pharmacological therapies used, including formulation, dosages, routes, and durations - Figures and other media are used to illustrate | Not applicable |
| 7d | **Operator details** – comprehensively describe:   - Requirement for additional training - Learning curve for technique - Relevant training, specialisation and operator’s experience (e.g. average number of the relevant procedures performed annually) | Not applicable |
| 7e | **Quality control** – comprehensively describe:   - Measures taken to reduce inter-operator variability - Measures taken to ensure consistency in other aspects of intervention delivery - Measures taken to ensure quality in intervention delivery | Not applicable |
| 7f | **Post-intervention considerations** – comprehensively describe:   - Post-operative instructions (e.g. avoid heavy lifting) and care - Follow-up measures - Future surveillance requirements (e.g. blood tests, imaging etc.) | Not applicable |
| 8 | **Outcomes** – comprehensively describe:   - Primary outcomes, including validation, where applicable - Secondary outcomes, where appropriate - Definition of outcomes - If any validated outcome measurement tools are used, give full reference - Follow-up period for outcome assessment, divided by group | Not applicable |
| 9 | **Statistics** – Data were reviewed for consistency, coding errors, completeness, accuracy, clarity, and missing values before being entered into Epi-Data Manager version 4.6.1 and analyzed by STATA version 16.0 Software. With the use of the median, mean, proportion, frequency, and interquartile range, descriptive and summary statistics were computed. Tables and graphics were used to present the data. The median time to frequent opportunistic infections over the follow-up period was estimated using the Kaplan-Meier curve, and log-rank tests were used to evaluate survival curves between various categories of predictive variables. The number of children who acquired common OIs throughout the follow-up period was divided by the number of person-years the children were under observation to determine the incidence of common opportunistic illnesses. The Schoenfeld residuals test (global test = 0.85200) was used to verify the basic premises of the Cox proportional hazard regression model, and the Cox-Snell residual was compared to the cumulative hazard function to determine the model's fit. To find predictors of typical opportunistic infections, the Cox proportional hazard model was fitted to both bivariable and multivariable data. To identify a significant variable, the bivariable analysis variables with a p-value of up to 0.25 were added to the multivariable model. Variables with p-values < 0.05 were regarded as statistically significant predictors of typical OIs in the final model. The presence and degree of correlations were summarized using an adjusted HR (AHR) with 95% confidence intervals. | 7&8 |
| RESULTS | | |
| 10a | **Participants** –  **Socio-demographic characteristics of the children**  The medical records of 354 HIV-infected children all were obtained. A completeness rate of 96.3 percent was achieved with the study of 341 medical records of HIV-infected children receiving ART. The median age of the study participants was 8 years (IQR = 4.5, 10). Two-thirds of 237 (69.5%) children were over 10 years old. More than half of the 180 (52.8%) children were males. The vast majority of children (83.6%) resided in cities, and 88.3% of them lived with their parents. Twenty-nine (8.5%) were government employees by occupation (Table 1). | 8 |
| 10b | **Participant comparison**   - Include table comparing baseline characteristics of cohort groups - Give differences, with statistical relevance - Describe any group matching, with methods | Not applicable |
| 10c | **Intervention** – comprehensively describe:   - Degree of novelty of intervention - Learning required for interventions - Any changes to interventions, with rationale and diagram, if appropriate | Not applicable |
| 11a | **Outcomes** – comprehensively describe:   - Clinician-assessed and patient-reported outcomes for each group - Relevant photographs and imaging are desirable - Any confounding factors and state which ones are adjusted | Not applicable |
| 11b | **Tolerance** – comprehensively describe:   - Assessment of tolerability of exposure/intervention - Cross-over with explanation - Loss to follow-up (fraction and percentage), with reasons | Not applicable |
| 11c | **Complications** – comprehensively describe:   - Adverse events and classify according to Clavien-Dindo classification* - Timing of adverse events - Mitigation for adverse events (e.g. blood transfusion, wound care, revision surgery etc.)   *Dindo D, Demartines N, Clavien P-A. Classification of Surgical Complications. A New Proposal with Evaluation in a Cohort of 6336 Patients and Results of a Survey. Ann Surg. 2004; 240(2): 205-213 | Not applicable |
| 12 | **Key results** –  **Clinical, laboratory, and medication-related characteristics**  CD4 counts, or percentages above the cutoff, were present in slightly more than half (53.1%) of the children. Two-thirds (71.3%) of children had a good degree of adherence to ART during the follow-up period, and around half (54.3%) of children were categorized as WHO clinical stages I and II. Seventy-two (21.1%) of the children experienced drug-related side effects, and 61.6% of the children had hemoglobin levels below 10 mg/dl. Additionally, IPT and CPT usage among youngsters was 72.7 percent and 45.7 percent, respectively. Approximately 89.4% of children took ART for more than 34 months, whereas 16.1% had unsuccessful medical therapy (Table 2).  **Incidence of common opportunistic infections during follow-up**  In this study 38.1% of children develop common OIs. The total OIs incidence rate was 6.0 (95 % CI: 5.0-7.1) per 100 child-years of observation. Participants in this study were observed for a minimum of 9 months and a maximum of 122 months, giving a total of 21,629 months or 1802.4 years of observation (figure 1). The median OIs-free survival duration was 97 months (IQR = 66, 117) in this study. The most frequent condition seen in 32.3 percent of the children was tuberculosis, which was followed by bacterial pneumonia (16.1 percent) and herpes zoster (12.3%) (Table3).  **Common opportunistic infections free survival time of predictor variable**  Compared to children with WHO clinical stages I and II, those with WHO clinical stages III and IV at the start of ART had a shorter OIs-free survival time (Figure 2). Children with mild immunodeficiency (CD4 count or percent above the threshold) had a longer OIs-free survival time than children who presented with severe immunodeficiency (CD4 count or percent below the threshold) (Figure 3). Children with low hemoglobin levels (<10 mg/dl) and those who did not take CPT also had shorter free OIs-free survival times than their counterparts (Figure 4) and (Figure 5).  **Predictors of common opportunistic infections**  CD4 count or %, WHO clinical staging, Duration of follow-up in months, weight for age,  taking IP prophylaxis, disclosure status, level of hemoglobin, level of adherence, taking CPT prophylaxis, and initiation regimen were variables included in the multivariable analysis. WHO clinical stages III and IV, CD4 levels or percentages below the threshold, CPT non-users, and low hemoglobin levels (<10 mg/dl) were discovered to be important predictors of OIs. The risk of having OIs was 1.77 times higher in children with WHO clinical stages III and IV (AHR: 1.77; 95% CI: 1.13, 2.77) than in children with WHO clinical stages I and II. Children with CD4 counts or percentages below the criteria were 2.53 times (AHR: 2.53; 95% CI: 1.65, 3.87) more likely to have OIs than children with CD4 counts or percentages above the threshold. Children who did not utilize CPT were 2.1 times (AHR: 2.10; 95% CI: 1.40, 3.08) more likely to acquire OIs than those who did use CPT. Finally, children with low hemoglobin levels (less than 10 mg/dl) were roughly 1.88 times (AHR: 1.88, 95% CI: 1.25, 2.82) more likely to develop OIs than children with normal hemoglobin levels (greater than 10 mg/dl) (Table 4).  Table 4: Cox-proportional hazard analysis of predictors of Common OIs among HIV-infected children on antiretroviral therapy at northeast Ethiopia public Hospitals, 2022   \| Variables \| \| Survival status \| \| CHR (95% CI) \| AHR (95% CI) \| \| --- \| --- \| --- \| --- \| --- \| --- \| \| Event \| Censored \| \| Age of the child (years) \| <5 years \| 38 \| 25 \| 1 \| - \| \| 5-9 years \| 29 \| 12 \| 1.02(0.51-1.57) \| - \| \| ≥10 years \| 144 \| 93 \| 1.18(0.75 -1.84) \| - \| \| Sex \| Male \| 110 \| 70 \| 1.11(0.78 -1.57) \| - \| \| Female \| 101 \| 60 \| 1 \| - \| \| CD4 count or % \| Below threshold \| 64 \| 96 \| 3.36(2.27-4.97) \| 2.53(1.65-3.87)** \| \| Above threshold \| 147 \| 34 \| 1 \| 1 \| \| WHO clinical staging \| I/II \| 155 \| 30 \| 1 \| 1 \| \| III/IV \| 56 \| 100 \| 3.47(2.30-5.23) \| 1.77(1.13-2.77)* \| \| IP \| Given \| 153 \| 95 \| 1 \| 1 \| \| Not given \| 58 \| 35 \| 1.37(0.92-2.04) \| 0.95(0.62-1.45) \| \| CPT \| Given \| 122 \| 34 \| 1 \| 1 \| \| Not given \| 89 \| 96 \| 2.53(1.71-3.74) \| 2.10(1.40-3.08)* \| \| Hemoglobin level \| <10 mg/dl \| 41 \| 90 \| 3.14(2.16-4.57) \| 1.88(1.25-2.82)** \| \| ≥ 10 mg/dl \| 170 \| 40 \| 1 \| 1 \| \| Weight for age \| Normal \| 187 \| 105 \| 1 \| 1 \| \| Underweight \| 24 \| 25 \| 1.37(0.88-2.13) \| 1.17(0.72-1.90) \| \| Height for age \| Normal \| 189 \| 111 \| 1 \| - \| \| Stunting \| 22 \| 19 \| 1.08(0.66 -1.76) \| - \| \| Disclosure status \| Disclosed \| 102 \| 72 \| 1 \| 1 \| \| Non disclosed \| 109 \| 58 \| 1.22(0.86 -1.72) \| 0.78(0.54-1.13) \| \| Adherence \| Good \| 162 \| 81 \| 1 \| 1 \| \| Fair /Poor \| 49 \| 49 \| 1.84(1.29 -2.63) \| 1.45(0.97-2.18) \| \| duration on ART \| <34 months \| 27 \| 9 \| 1 \| 1 \| \| >34 months \| 184 \| 121 \| 3.57(1.77-7.20) \| 1.20(0.93-3.44) \| \| Initiation regimen \| EFV based \| 65 \| 40 \| 1.38(0.94 -2.00) \| 1.31(0.89-1.94) \| \| NVP,PI and other based \| 146 \| 90 \| 1 \| 1 \|   **Notice** - *Significant at <0.05 ** Significant at <0.01; CHR: Crude hazard ratio; AHR: adjusted hazard ratio; 1: reference category; CI: confidence interval CPT: cotrimoxazole prophylactic therapy; IPT: isoniazid prophylactic therapy;HGB | 8,9&20 |
| DISCUSSION | | |
| 13 | **Discussion** – A multicenter retrospective follow-up study was conducted in northeast Ethiopian public hospitals to investigate the incidence and predictors of common opportunistic illnesses among HIV-infected children on ART.  In study the median common OIs-free survival time was 97 months, and the overall incidence rate was 6.0 (95% CI: 5.0-7.1) per 100 child-years of observation among HIV-infected children in public hospitals in northeast Ethiopia. This finding is consistent with research undertaken in Northwest Ethiopia (5.3 per 100 person-years) (8) Italy (6.76 per 100 person-years) (14), and the United States(4.99 per 100 person-years) (15). However, the incidence of common OIs discovered in this study is higher than that seen in Latin America (1.1 per 100 person-years) (16) and Brazil (2.63 per 100 person-years) (17). The reasons for this could include the variance in follow-up times, the criteria for outcome variables, and the study population's inclusion of both adults and children. Furthermore, this disparity may be explained by the fact that affluent countries have more sophisticated procedures for the early diagnosis, treatment, and management of OIs than resource-constrained settings like Ethiopia, and the larger challenges of poverty, overcrowding, and malnourishment in developing countries may be a factor in the higher frequency of OIs among HIV-infected children.  During the follow-up period, tuberculosis was the most common opportunistic illness (32.3%). This finding is congruent with research undertaken in Ethiopia (29.8%) and India (34.6%) (18, 19). In contrast, a study conducted in Ethiopia, North America, Latin America, and China discovered that pneumonia is a prevalent opportunistic infection (8, 15, 20-22). Furthermore, in this study, chronic diarrhea accounted for 16.1% of all common OIs.  In this study, the probability of acquiring OIs was 1.77 times higher in children with WHO clinical stages III and IV than in children with WHO clinical stages I and II. This outcome is consistent with research conducted in Ethiopia (18), India (23, 24), and Asia (25) . It is explained by the fact that advanced disease weakens immunity, resulting in increased viral multiplication and higher loads of opportunistic infections.  Children with CD4 counts or percentages below the criteria had a 2.53 times (AHR: 2.53; 95 percent CI: 1.65, 3.87) higher risk of developing OIs than children with levels above the threshold. This discovery is consistent with research conducted in Ethiopia (18), Uganda (26), India (19), and Asia (25). CD4 cells are crucial components of the immune system because they help the body fight infections. Therefore, any conditions that lower CD4 cell counts will weaken the immune system of children with HIV who are vulnerable to the development of opportunistic infections.  The risk of OIs was 1.88 times higher in children with low hemoglobin levels children with normal hemoglobin levels. This research is backed up by studies undertaken in Ethiopia (8), Nigeria (27), and Uganda (26). This is because low hemoglobin levels can have serious effects for those living with HIV, ranging from impaired productivity and performance to a relationship to disease process and increased death (28).  Children who did not utilize CPT were 2.1 times more likely to develop OIs than those who did use CPT. This conclusion is consistent with research conducted in Ethiopia(8, 18), Zambia (29), and Latin America (16). Cotrimoxazole preventive therapy (CPT) is a realistic, inexpensive, and well-tolerated approach of employing cotrimoxazole intervention for HIV/AIDS patients to reduce HIV/AIDS-related comorbidities and deaths caused by various bacteria, fungi, and protozoa. Furthermore, the Ethiopian ART guideline recommends starting CPT early in HIV-positive children who will benefit from it in order to prevent OIs (10, 30). | 9,10&11 |
| 14 | **Strengths and limitations** –  **Limitation of the Study**  One of the study's limitations is its retrospective nature. As a result, clinically relevant predictor variables such as children's educational status and family economic status, as well as community hygiene practices and patients' and caregivers' awareness levels were omitted from this study. | 11 |
| 15 | **Relevance and implications**- I recommend that researchers conduct this study prospectively. | 11 |
| CONCLUSION | | |
| 16 | **Conclusions**  In comparison to other research, the incidence rate of opportunistic infections among HIV-infected children was shown to be high in this study. Opportunistic infection rates were linked to low hemoglobin levels (<10 mg/dl), low CD4 counts or percentages, WHO clinical stages III and IV, and non-users of CPT. As a result, several tactics, approaches, and programs should be considered in order to lower the occurrence of opportunistic infections among HIV-positive children. It is particularly critical to stress the predictors of opportunistic infections among children revealed in this study. More research is needed to find additional factors impacting the high occurrence of opportunistic infections. | 11 |
| DECLARATIONS | | |
| 17a | **Conflicts of interest**  The authors declare no conflict of interest. |  |
| 17b | **Funding**  This study did not receive any specific grant from funding agencies in the public, commercial, or not-for-profit sectors. |  |
| 17c | **Contributorship**  Endalk Birrie Wondifraw, Birhanu Desu Tefera, Mulusew Zeleke, Samuel Nebyu ; participate in writing proposal, analysed the data, wrote the result and discussion. Lehulu Tilahun Mulugeta W/Selassie, **Zenebe Tefera** ; participate in analyzing the data, writing result and prepared manuscript. |  |

*STROCSS 2021 guidelines apply to cohort studies as well as other observational studies
